# Concomitant Atrial Fibrillation Procedures During Cardiac Surgery in a UK Center: Reflection of Worldwide Practice?

**DOI:** 10.3389/fcvm.2022.780893

**Published:** 2022-03-10

**Authors:** Alina-Adriana Mistirian, Martin T. Yates, Wael I. Awad

**Affiliations:** Department of Cardiothoracic Surgery, St Bartholomew's Hospital, London, United Kingdom

**Keywords:** atrial fibrillation, concomitant ablation, cardiac surgery, Cox-Maze, left atrial appendage occlusion

## Abstract

**Background:**

Guidelines recommend concomitant atrial fibrillation (AF) ablation during cardiac surgery to restore normal sinus rhythm (NSR). The study determines, to what extent patients with AF undergoing cardiac surgery at our institution received a concomitant AF procedure, what these procedures entailed, and short-term outcomes.

**Methods:**

A retrospective study of 2,984 patients undergoing cardiac surgery over 18 months. Patients who were in preoperative AF were identified and those who underwent a concomitant AF procedure (Group 1) were compared with those who did not (Group 2).

**Results:**

Three hundred and thirteen (10.5%) patients had pre-operative AF; paroxysmal (19.5%), persistent (11.8%), longstanding (63%), unknown (5.8%). 116/313 (37.1%) patients had a concomitant AF procedure: 7.7% patients had a concomitant AF ablation and 29.4% had only a Left Atrial Appendage Occlusion (LAAO). Fewer patients with paroxysmal and persistent AF underwent concomitant AF procedures compared with the ones who had no AF procedures (6.7 vs. 12.8% and 17.6 vs. 31%, respectively). Greater in-hospital survival (99.1 vs. 93.9%, *p* = 0.025) and survival at a mean follow up of 6 weeks (97.4 vs. 89.3%, *p* = 0.09) was probably determined by patient's preoperative comorbidities. There were no differences in readmission rates, permanent pacemaker insertion, cerebral events or NSR at discharge or follow-up, between groups.

**Conclusions:**

In our center, concomitant AF ablation is performed only in 7.7% of cases, 29.4% had only an LAAO performed at the time of surgery. There was no difference in restoring NSR, cerebral events, or readmission rates compared with patients who had nothing done for their preoperative AF.

## Introduction

Atrial fibrillation (AF) is a common supraventricular arrhythmia, with increased prevalence with increasing age. The incidence of AF is greater in patients who have significant coronary artery disease or valvular heart pathology (1). AF is associated with a five-fold risk of stroke, a three-fold incidence of congestive heart failure, higher hospitalization and mortality (2). It significantly impairs patients' quality of life and reduces long-term survival. Management strategies include rate and rhythm control, anticoagulation following risk assessment for stroke and bleeding, and AF ablation.

AF ablation can be performed as a stand-alone surgical procedure, on a beating heart, via thoracotomy or sub-xiphoid approach or using cardio-pulmonary bypass, through mini or full midline sternotomy or concomitantly with other cardiac surgery, with good results reported (2). Concomitant AF ablation can vary from pulmonary vein isolation to a bi-atrial ablation or a full Cox-Maze procedure. The Cox-Maze (CM) procedure is considered the gold standard surgical AF ablation technique. Clinical results have shown that the CM IV achieves equivalent success rate of the original CM procedure while significantly reducing operative time and lowering complication rates (3).

Left atrial appendage occlusion (LAAO) in patients with AF, although not an ablation procedure has been shown to reduce perioperative stroke and the long-term risks of thromboembolic complications (4). The most recent 2017 guidelines from the Society of Thoracic Surgeons (STS) recommend concomitant AF ablation for patients undergoing all types of cardiac procedures but particularly during mitral valve (MV) surgery, with good results in restoring normal sinus rhythm (NSR) (5).

There is increasing evidence demonstrating reduced stroke rates (6), fewer bleeding incidents (7), fewer readmissions for AF or heart failure (8, 9), but also improved long term survival and quality of life (10, 11), for patients who have a concomitant AF ablation procedure. The best outcomes following concomitant AF ablation during cardiac surgery depend on good patient selection. Higher chances in restoring NSR after concomitant AF ablation, are seen in patients with preoperative AF duration <1 year (paroxysmal or persistent) (12). Other studies show that the echocardiographic dimensions of the left atrium (LA) are also important to determine the chances of restoring normal sinus rhythm, with best results achieved if LA dimensions are <55 mm (13). Other factors such as diabetes, hypertension, or smoking are associated with worse results after AF ablation (13, 14).

Despite strong evidence for concomitant AF ablation, surgeons are still reluctant to offer this treatment during cardiac surgery for a variety of reasons including increased operative time, the need to open the left or right atria and concerns regarding intraoperative complications. However, patients may be missing a unique opportunity to have their AF treated at the time of their cardiac surgery.

The aim of this study is to determine to what extent patients with AF undergoing cardiac surgery at our institution received a concomitant AF procedure, what these procedures entailed, and whether these procedures are adequate in restoring NSR and maintaining it at short term follow up. Through this study we aim to raise awareness about the potential advantage of concomitant AF ablation during cardiac surgery. Finally, we aim to provide some recommendations to improve the management of patients with atrial fibrillation undergoing cardiac surgery.

## Methods

A retrospective study of all patients who underwent cardiac surgery at our institution between June 2017 and January 2019. Our hospital is a large tertiary cardiac center undertaking around 2,500 cardiac cases per annum. Study cohort were patients presenting with preoperative AF. The types of AF were stratified further and defined as following: (i) Paroxysmal AF- recurrent AF (≥2 episodes) that terminates spontaneously within 7 days or episodes of AF ≤48 h duration, ended with electrical or pharmacologic conversion; (ii) Persistent AF- continuous AF, sustained beyond 7 days or episodes of AF in which the decision of electrical or pharmacological cardioversion is made after ≥48 h, but prior to 7 days; (iii) Longstanding persistent AF- continuous AF of >12 months (15).

Patients with preoperative AF were divided into two groups: Group 1 patients underwent a concomitant AF procedure at the time of cardiac surgery and Group 2 patients did not. The two groups were compared with respect to patient characteristics and comorbidity, postoperative complications, hospital stay, and early outcomes (in-hospital and at routine follow-up at 6–8 weeks following discharge from hospital).

*Definitions:* severe renal impairment: patient on dialysis or creatinine clearance <30 mls/min; extracardiac arteriopathy: claudication, carotid occlusion or >50% stenosis, amputation for arterial disease or previous or planned intervention on the abdominal aorta, limb arteries, or carotids; chronic pulmonary disease: long term use of bronchodilators or steroids for lung disease; pulmonary hypertension: systolic pressure >55 mg Hg.

The following procedures were considered as AF ablation procedures: Cox-Maze procedure, pulmonary vein (PV) isolation (radiofrequency or cryoablation) and left atrium (LA) ablation (radiofrequency or cryoablation). We included left atrial appendage occlusion (LAAO) as an AF procedure and not an ablation.

We recorded patient's rhythm before the surgical procedure, postoperatively, at discharge from hospital and in the out-patient clinic, 6–8 weeks from discharge. The ECG was the main tool used to record the rhythm. Only a few patients had a 24 h ECG at follow up review. The duration of preoperative AF was documented from patient's medical records.

Continuous variables were expressed as mean ± 1 standard deviation. Differences between the two Groups were analyzed with Chi-squared test and *T*-test for Independent Means. A *p* < 0.05 was considered significant.

## Results

A total of 2,984 patients underwent a cardiac surgical procedure over this 18 month period at our institution. Three hundred and thirteen (10.4%) had preoperative AF: 61 (19.5%) paroxysmal, 37 (11.8%) persistent, 45 (14.4%) longstanding, 152 (48.5%) permanent, and 18 (5.8%) could not be classified. Table 1 shows the characteristics of Group 1 and Group 2 patients with preoperative AF. Overall the characteristics of the two groups of patients were similar, except for two of them: the presence of infective endocarditis (0.9 vs. 6.1%, *p* = 0.025) and previous cardiac surgery (1.7 vs. 14.2%, *p* = 0.001), both significantly higher in Group 2 patients.

**Table 1:** Patients characteristics.

	**Group 1: AF procedure** ***n*** **= 116**	**Group 2: No AF procedure** ***n*** **= 197**	***P*** **-value**
Male gender	70 (60.3%)	123 (62.4%)	0.713
Age at operation (years)	71 ± 9.67	70 ± 10.39	0.984
Severe renal impairment	27(23.3%)	59 (29.9%)	0.206
**Active IE**	**1 (0.9%)**	**12 (6.1%)**	**0.025**
Extracardiac arteriopathy	3 (2.6%)	11 (5.6%)	0.215
Chronic pulmonary disease	17 (14.6%)	26 (13.2%)	0.717
MI within 30 days	5 (4.3%)	8 (4.1%)	0.914
Previous TIA or CVA	18 (15.5%)	20 (10.1%)	0.160
IDDM	2 (1.7%)	8 (4.1%)	0.256
HTN	90 (77.6%)	158 (80.2%)	0.581
Smoking history	6 (5.2%)	13 (6.6%)	0.609
Pulmonary HTN	23 (19.8%)	54 (27.4%)	0.132
Poor LV function preop (LVEF <30%)	6 (5.2%)	19 (9.6%)	0.158
NYHA III/IV preop	19 (16.4%)	23 (11.7%)	0.238
Preop PPM	6 (5.2%)	14 (7.1%)	0.499
**Previous cardiac surgery**	**2 (1.7%)**	**28 (14.2%)**	**<0.001**

*IE, infective endocarditis; MI, myocardial infarction; IDDM, insulin-dependent diabetes mellitus; HTN, hypertension; TIA, transient ischemic attack; CVA, cerebrovascular accident; LV, left ventricle; NYHA, New York Heart Association classification; PPM, permanent pacemaker. Bold value indicate that the difference between the values was statistically significant*.

Table 2 shows the type of AF in both Group 1 and Group 2 patients. The majority 77 (66.4%) of patients in Group 1 (who had a concomitant AF procedure performed) mainly presented with longstanding AF. Only 27 (31%) of patients who presented with AF duration <1 year before cardiac surgery (paroxysmal and persistent) had a concomitant AF procedure (ablation procedure or just a LAAO).

**Table 2:** Type of AF in group 1 and group 2 patients.

**AF type**	**Group 1: AF procedure** ***n* = 116**	**Group 2: No AF procedure** ***n* = 197**	***P*** **-value**
Paroxysmal 61 (19.5%)	21 (18.1%)	40 (20.3%)	0.634
Persistent 37 (11.8%)	15 (12.9%)	22 (11.2%)	0.640
Longstanding 197 (62.9%)	77 (66.4%)	120 (60.9%)	0.333
Unknown 18 (5.8%)	3 (2.6%)	15 (7.6%)	—

*AF, atrial fibrillation*.

**Figure 1:**
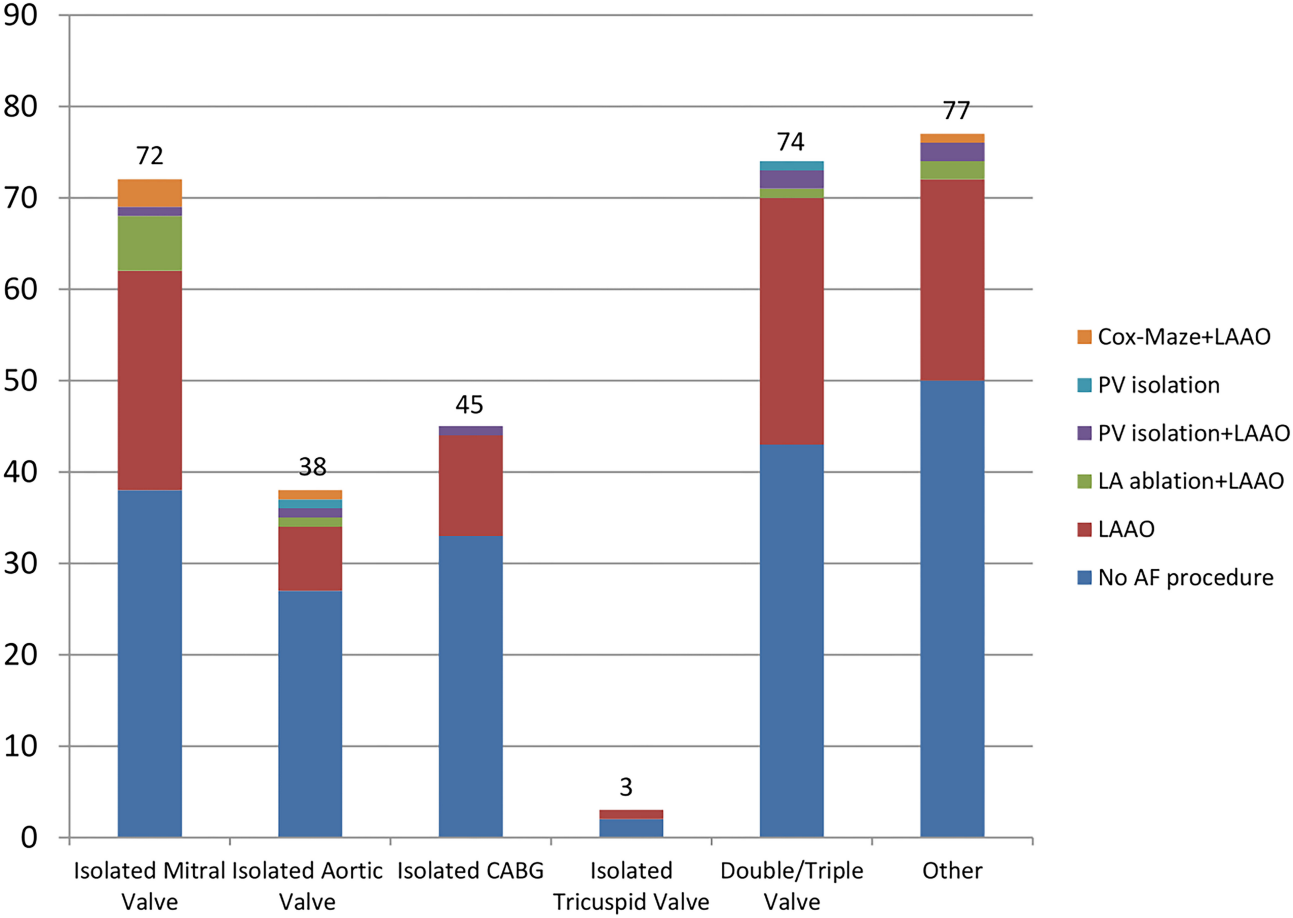
Cardiac procedures and concomitant AF procedures performed. AF, atrial fibrillation; PV, pulmonary vein; LAAO, left atrial appendage occlusion; LA, left atrium; CABG, coronary artery bypass graft; “Other” procedures category includes: Valve replacement + CABG, Valve replacement + septal myectomy, Ascending aorta/hemiarch replacement + CABG/valve replacement, myxoma + CABG.

Table 3 illustrates the types of cardiac procedures which the patients included in Group 1 underwent. The cardiac procedures performed for patients included both in Group 1 and Group 2 were similar. Most commonly performed procedures were: isolated valve replacement/repair (aortic/mitral), double or triple valve replacement, coronary artery bypass graft surgery (CABG), major aortic surgery (aortic root replacement, ascending aorta, or hemiarch replacement) and combined procedures. Of those patients undergoing a concomitant atrial fibrillation procedure, 92 (79%) underwent isolated left atrial appendage occlusion. Two (1.7%) further patients had isolated pulmonary vein isolation. Twenty two (19%) patients who had left atrial appendage occlusion combined with an ablation procedure as follows: Cox-Maze 5 (4.3%), LA ablation 10 (8.6%) and PV Isolation 7 (6%). Table 4 shows the exact AF procedures undertaken in relation to the type of AF: paroxysmal, persistent, and longstanding. Figure 1 shows the type of concomitant AF procedures undertaken at the time of the respective cardiac procedure performed.

**Table 3:** Cardiac procedures performed in n=116 patients undergoing concomitant AF procedure.

**Isolated procedures**				**Mixed procedures**						
**CABG**	**Valve repair/replacement**			**Double/triple valve procedures**			**Other procedures**			
***n*** **= 12**	***n*** **= 46**			***n*** **= 31**			***n*** **= 27**			
	Mitral *n* = 34	Aortic *n* = 11	Tricuspid *n* = 1	AVR+ MVR/+ TV repair *n* = 10	MVR +TV repair *n* = 14	MV repair +TV repair/+ AVR *n* = 7	VR/ repair + CABG *n* = 16	VR+ SM *n* = 5	AAR/hemiarch+ VR/CABG *n* = 4	AM + CABG *n* = 2

*AAR, ascending aorta replacement; AM, atrial myxoma; CABG, coronary artery bypass graft; AVR, aortic valve replacement; MVR, mitral valve replacement; MV, mitral valve; SM, septal myectomy; TV, tricuspid valve; VR, valve replacement*.

**Table 4:** Concomitant AF procedure performed in different types of AF.

**AF type**	**Concomitant AF procedure *n* = 116**				
	**Cox-Maze + LAAO**	**LAa + LAAO**	**PVI + LAAO**	**PVI**	**LAAO**
	***n*** **= 5**	***n*** **= 10**	***n*** **= 7**	***n*** **= 2**	***n*** **= 92**
Paroxysmal	2 (1.7%)	3 (2.6%)	2 (1.7%)	1 (0.9%)	13 (11.2%)
Persistent	2 (1.7%)	1 (0.9%)	0	0	12 (10.3%)
Longstanding	1 (0.9%)	5 (4.3%)	5 (4.3%)	1 (0.9%)	65 (56%)
Unknown	0	1 (0.9%)	0	0	2 (1.7%)

*AF, atrial fibrillation; LAa, left atrial ablation; LAAO, left atrial appendage occlusion; PVI, pulmonary vein isolation*.

Patients in Group 1 and Group 2 were classified into three categories based on preoperative cardiac echocardiography dimensions of the left atrium [dilated (LA < 55 mm), significantly dilated (LA > 55 mm) and severely dilated (≥65 mm)] and the preoperative duration of AF as illustrated in Table 5. Of 79 patients with longstanding AF and severely dilated LA, 37 (46.8%) patients had a concomitant AF procedure and 42 (53.2%) did not (*p* = 0.987).

**Table 5:** LA dimensions and type of AF in group1 vs. group 2 patients.

**AF type**	**Preoperative LA dimensions**								
	**Dilated**			**Significantly dilated**			**Severely dilated**		
	**(<55 mm)**			**(>55 mm)**			**(≥65mm)**		
	**Group 1**	**Group 2**	***P*** **=**	**Group 1**	**Group 2**	***P*** **=**	**Group 1**	**Group 2**	***P*** **=**
	***n*** **(%)**	***n*** **(%)**		***n*** **(%)**	***n*** **(%)**		***n*** **(%)**	***n*** **(%)**	
Paroxysmal	9 (2.9)	25 (8)	0.442	2 (0.6)	3 (1)	0.701	6 (1.9)	6 (1.9)	0.813
Persistent	5 (1.6)	6 (1.9)	0.300	3 (1)	7 (2.2)	0.845	7 (2.2)	6 (1.9)	0.586
Longstanding	22 (7)	44 (14)	0.651	7 (2.2)	16 (5.2)	0.745	**37 (4.2)**	**42 (1.6)**	**0.987**
Unknown	1 (0.3)	5 (1.6)	0.418	1 (0.3)	1 (0.3)	0.586	1 (0.3)	4 (1.3)	0.219

*LA, left atrium; AF, atrial fibrillation. Bold value indicate that the difference between the values was statistically significant*.

Postoperative complications in both patient groups are shown in Table 6. Group 1 patients had a significantly lower return to theater compared with Group 2 patients (2.6 vs. 9.1%, *p* = 0.026), a shorter hospital stay (11.9 ± 6 vs. 16.6 ± 19, *p* = 0.013) and better in-hospital survival (99.1 vs. 93.9%, *p* = 0.025). The majority of patients were discharged on antiarrhythmic medication (amiodarone, beta-blockers, or calcium channels blockers), except for a few cases where patients were bradycardiac and could not be discharged on any antiarrhythmic medications. Group 1 patients were more likely to be discharged on antiarrhythmic drugs compared with Group 2 patients, 85.3 vs. 73.1%, *p* = 0.012), respectively.

**Table 6:** In-hospital outcomes.

	**Group 1: concomitant AF procedure** ***n*** **= 116**	**Group 2: no AF procedure** ***n*** **= 197**	***P*** **=**
**Return to theater**	**3 (2.6%)**	**18 (9.1%)**	**0.026**
Mean ITU LOS (nights)	4.7 ± 6	6.5 ± 11	0.120
Post-op cerebral events	3 (2.6%)	12 (6.1%)	0.161
Post-op PPM insertion	16 (13.8%)	29 (14.7%)	0.821
**Mean hospital LOS (days)**	**11.9 ±6**	**16.6 ±19**	**0.013**
NSR at discharge	35 (30.2%)	61 (31%)	0.883
**In-hospital survival**	**115 (99.1%)**	**185 (93.9%)**	**0.025**

*Continuous variables are listed as mean ± standard deviation; AF, atrial fibrillation; n, number; P, p-value; ITU, intensive therapy unit; LOS, length of stay; post-op, postoperative; PPM, permanent pacemaker; NSR, normal sinus rhythm. Bold value indicate that the difference between the values was statistically significant*.

In Group 1, patients who had an AF procedure done, 114/116 pts (98.3%) had a LAAO.

In 79/114 pts (69.3%) with LAAO the rhythm identified at discharge was AF. The rhythm identified for the other 2 pts with isolated PVI at discharge was AF, as well. Overall, in Group 1 the rate of postoperative AF at discharge was 69.8%. Looking at Group 2, 110/197 pts (55.8%) were discharged in AF. Postoperative AF was significantly higher at discharge for Group 1 compared with Group 2 (69.8 vs. 55.8%, *p* = 0.01).

In addition to antiarrhythmic therapy, patients were discharged on oral anticoagulant therapy [warfarin or direct oral anticoagulants (DOACs)]. The majority [74 (68%)] of patients without a mechanical valve replacement and in AF at discharge were discharged on DOACs. Patients undergoing an AF procedure (even just an LAAO procedure) were more likely to be discharged on anticoagulation therapy compared with those who did not (93.9 vs. 82.2%, *p* = 0.033).

277/313 patients (88.5%) had a mean follow up of 6.09 ± 1.57 weeks, excluding the 22 patients who died and another 14 patients who were lost to follow up. Patients in Group 1 with a concomitant AF procedure had better survival compared with patients in Group 2, although this did not reach significance (97.4 vs. 89.3%, *p* = 0.09). There were no differences between the two Groups in the incidence of normal sinus rhythm (58.7 vs. 56.9%, *p* = 0.759) or hospital readmissions for AF or anticoagulation issues (7.7 vs. 6%, *p* = 0.569), at follow up.

At follow up, 47/114 pts (41.2%) with LAAO were identified as being in AF, and overall 41.4% pts were in AF in Group 1. In Group 2, 73/197 pts (37.1%) were identified as being in AF. There is a small difference between the 2 groups with more pts with postoperative AF at follow up in Group 1 (41.4 vs. 37.1%, *p* = 0.44).

## Discussion

Concomitant AF ablation during cardiac surgery is strongly supported by the most recent 2017 guidelines from the STS. Studies confirm that restoring NSR through concomitant AF ablation reduces the risk of stroke (5), bleeding (6), rate of readmissions for AF (7), risk of heart failure in addition to improving long term survival of patients and their quality of life (8–11). We have shown that adherence to these guidelines remains low, even in a high volume cardiac surgery center. In our study, out of 313 patients who presented with preoperative AF, and undergoing cardiac surgery, 37% had an AF procedure performed, of whom only 7.7% had an AF ablation. The majority of patients having a concomitant AF procedure only had a LAA clip, despite evidence showing that when a more complex ablation procedure was performed, there was a higher chance of restoring NSR and improved outcomes.

Despite evidence-backed guidelines regarding the safety and efficacy of concomitant AF ablation, this was not routinely performed in our cohort of patients. One frequent explanation provided for this is that in the presence of comorbidities, the risk to the patient is increased. In our study, both Groups (patients who had a concomitant AF ablation and those who did not) were similar regarding patient characteristics and co-morbidity, except the presence of preoperative infective endocarditis and previous cardiac surgery, which are both indeed associated with increased procedure risks. Hypertension, diabetes, and smoking are major independent predictors for AF ablation failure (1), however this did not appear to play a role in selection of our patients (Table 1).

Patients undergoing all types of cardiac surgery will benefit from a concomitant AF ablation, but those undergoing procedures on the mitral valve are most likely to benefit and given that the left atrium is opened, no extra incisions are required (5). In our study, 34 patients with preoperative AF and undergoing an isolated MV procedure had a concomitant AF procedure however only three had a full Cox-Maze procedure. Further investigation would be required to understand why this group of patients does not gain the benefit of an ablation.

Concomitant AF ablation is effective restoring sinus rhythm in patients undergoing aortic valve replacement (AVR) or coronary artery bypass surgery (CABG), only one patient had a concomitant Cox-Maze procedure performed in this cohort of our study (16). This may be explained by reluctance to open the heart purely to perform an ablation.

Preoperative AF duration and left atrial dimensions predict the time until AF recurrences after concomitant left atrial ablation (17). The authors report that the sinus rhythm conversion rate was superior when preoperative AF duration was 2 years or less. Forlani et al. report that preoperative AF <1 year (paroxysmal/ persistent) is more likely to convert back to sinus rhythm after AF ablation (11). In our study, of the 98 patients most likely to benefit from a concomitant AF procedure (with paroxysmal / persistent AF), only one third had a concomitant AF procedure performed, and paradoxically, more than half of the patients with longstanding AF (patients who are least likely to benefit) had a concomitant AF procedure. The nature of an individual's AF history together with education of the cardiologist and surgeon is critical to perioperative decision making.

Preoperative LA dimensions predict AF recurrence after an ablation procedure (18). LA dimensions <55 mm predict a higher rate of success in restoring NSR after a concomitant AF ablation. In our patients with preoperative AF duration <1 year and LA dimensions <55 mm only a third had a concomitant AF procedure. Thus, almost 70% of the patients with the greatest chance of maintaining NSR after concomitant AF procedure did not have specific treatment for their AF. Pecha et al. (18) showed that the duration of AF and a preoperative smaller left atrial diameter are statistically significant predictors of long term ablation success. The authors investigated whether performing a concomitant AF ablation on patients with an enlarged LA >55 mm is successful and showed freedom from AF in 64.4% of patients at 1-year follow-up. However, they concluded that these patients need more interventions (medical or electrical cardioversion) and additional catheter-based ablation to achieve satisfactory results.

In our study, both Group 1 and Group 2 patients had similar rates of sinus rhythm at discharge and at short-term follow-up. More patients who had an AF procedure (98.3% had a LAAO) performed for their preoperative AF (Group 1) compared with those who had noting done, presented postoperative AF at discharge, as was highlighted by Yao et al. (19). Similar incidences of postoperative cerebral events, PPM insertion, and readmissions for AF or anticoagulation issues were also observed in both Groups. These findings may be explained by inconsistent patient selection and incomplete AF ablation (a full Cox-Maze was performed in only 4.3% of cases and majority of patients didn't have an AF ablation performed, but only a clip on their LAA), but does not address the effectiveness of the AF ablation procedures.

Patients who had an AF procedure performed were less likely to return to theater, had a shorter hospital stay, and improved survival. These findings can be explained by the patient's selection and inclusion in our cohort group. The patients included in Group 1, who had an AF procedure performed, were identified as lower risk for surgery compared with Group 2, which may explain the shorter hospital stay and improved survival, related to their comorbidities and not to have or not an AF procedure performed.

There are multiple ways to encourage surgeons to perform concomitant AF ablation in addition to education of the benefits as per international guidelines. This study, for example, was performed in a public health system where concomitant ablation attracts no additional reimbursement. Adherence to guidelines can be encouraged with increased reimbursement for additional procedures of proven benefit, such as AF ablation and application of a LAAO device, or withholding of reimbursement from those who do not follow guidelines or at least recommendations by a multi-disciplinary heart team.

## Study Limitations

During data collection, we encountered limited information in some patients' records regarding the duration of the preoperative AF and were, thus, unable to classify the type of AF. There was an absence of standardized echocardiography reporting with respect to LA dimensions, as our patient population came from many different referring centers, making the pre-operative assessment of LA size and the preoperative selection of patients for AF ablation, variable. In addition, our patient follow up has been incomplete, with only a small number of patients having a 24 h tape ECG to determine rhythm, some just a 12 lead ECG in out-patient clinic and some only a clinical determination of the heart rhythm and review by a trainee surgeon.

Our small cohort size does not allow for adjusted comparisons such as with propensity matching which requires large samples, with substantial overlap between treatment and control groups.

## Conclusions

Despite strong recommendations for, concomitant AF ablation is still not undertaken in most patients with preoperative AF undergoing cardiac surgery (in our center, AF ablation is performed in 7.7% of cases). The patients from both groups had similar rates of sinus rhythm at discharge and at short-term follow-up, but also similar incidences of postoperative cerebral events and readmissions for AF, which may be explained by inconsistent patient selection and incomplete AF ablation.

## Recommendations

As a consequence of these findings, we have made and implemented the following recommendations: establishing a dedicated multidisciplinary team meeting attended by cardiac electrophysiologists, general cardiologists and cardiac surgeons; identified a group of surgeons dedicated to and experienced in the surgical management of AF to champion this treatment; establishment of an AF register to improve the accuracy of patient data collection; standardized the reporting of cardiac investigations and the post-operative follow-up of patients undergoing AF procedures; employment of a specialist AF nurse.

## Data Availability Statement

The original contributions presented in the study are included in the article/supplementary material, further inquiries can be directed to the corresponding author.

## Ethics Statement

The studies involving human participants were reviewed and approved by Barts Health Clinical Effectiveness Unit Audit ID 10421 11/07/2019. The Ethics Committee waived the requirement of written informed consent for participation.

## Author Contributions

A-AM, MY, and WA have contributed in different but equal ways to this work. All authors contributed to the article and approved the submitted version.

## Conflict of Interest

The authors declare that the research was conducted in the absence of any commercial or financial relationships that could be construed as a potential conflict of interest.

## Publisher's Note

All claims expressed in this article are solely those of the authors and do not necessarily represent those of their affiliated organizations, or those of the publisher, the editors and the reviewers. Any product that may be evaluated in this article, or claim that may be made by its manufacturer, is not guaranteed or endorsed by the publisher.
